# Two-phase survey on the frequency of use and safety of MRI for hearing implant recipients

**DOI:** 10.1007/s00405-020-06525-3

**Published:** 2021-03-31

**Authors:** Paul van de Heyning, Griet Mertens, Vedat Topsakal, Ruben de Brito, Wilhelm Wimmer, Marco D. Caversaccio, Stefan Dazert, Stefan Volkenstein, Mario Zernotti, Lorne S. Parnes, Hinrich Staecker, Iain A. Bruce, Gunesh Rajan, Marcus Atlas, Peter Friedland, Piotr H. Skarzynski, Serafima Sugarova, Vladislav Kuzovkov, Abdulrahman Hagr, Robert Mlynski, Joachim Schmutzhard, Shin-Ichi Usami, Luis Lassaletta, Javier Gavilán, Benoit Godey, Christopher H. Raine, Rudolf Hagen, Georg M. Sprinzl, Kevin Brown, Wolf-Dieter Baumgartner, Eva Karltorp

**Affiliations:** 1 ENT Department, Antwerp University Hospital (UZA), Edegem/Antwerp, Belgium; 2 Hospital for Rehabilitation of Cranio-Facial Anomalies, Bauru-Sao Paulo, Brazil; 3 Department for ENT, Head and Neck Surgery, Bern University Hospital, Bern, Switzerland; 4 Bochum St. Elisabeth University Hospital, Bochum, Germany; 5 Córdoba Sanatorium Allende, Servicio de Otorrinolaryingologia (Servicio ORL), Córdoba, Argentina; 6 London Health Sciences Center-University Hospital, London, Ontario Canada; 7 Department of Otorinolaryngology, Kansas University Center for Hearing and Balance Disorders, Kansas City, USA; 8 Pediatric ENT Department, Royal Manchester Children’s Hospital, Manchester University NHS Foundation Trust, Manchester Academic Health Science Centre, Manchester, UK; 9 Division of Infection, Immunity and Respiratory Medicine, Faculty of Biology, Medicine and Health, University of Manchester, Manchester, UK; 10 University of Western Australia, Crawley, Perth, Australia; 11 Ear Sciences Center, Lions Hearing Clinic, Subiaco, Australia; 12 Institute of Sensory Organs, Nadarzyn/Kajetany, Poland; 13 Department of Teleaudiology and Screening, World Hearing Center of the Institute of Physiology and Pathology of Hearing, Kajetany, Poland; 14 St. Petersburg ENT and Speech Research Institute, St. Petersburg, Russia; 15 King Abdullah Ear Specialist Center, King Saud University Medical City, King Saud University, Riyadh, Saudi Arabia; 16 Universität Rostock “Otto Körner”, Klinik und Poliklinik für Hals-Nasen-Ohrenheilkunde, Rostock, Germany; 17 ENT Department, Medical University of Innsbruck, Innsbruck, Austria; 18 Shinshu University School of Medicine, Matsumoto, Japan; 19 Madrid Hospital La Paz, Madrid, Spain; 20 Centre Hospitalier Universitaire (CHU) de Rennes, Rennes, France; 21 Bradford Royal Infirmary Yorkshire Auditory Implant Center, Bradford, UK; 22 Würzburg ENT University Hospital, Würzburg, Germany; 23 St. Pölten University Hospital, St. Pölten, Austria; 24 UNC Ear and Hearing Center at Chapel Hill School of Medicine, Chapel Hill, NC USA; 25 Vienna Medical University-General Hospital AKH, Vienna, Austria; 26 Karolinska University Hospital, Solna, Sweden; 27 Present Address: Department of Otolaryngology, Head and Neck Surgery, Luzerner Kantonsspital, Luzern, Switzerland

**Keywords:** Safety, Magnetic resonance imaging, Auditory brainstem implant, Cochlear implant, Bone conduction, Middle ear implant

## Abstract

**Purpose:**

Magnetic resonance imaging (MRI) is often used to visualize and diagnose soft tissues. Hearing implant (HI) recipients are likely to require at least one MRI scan during their lifetime. However, the MRI scanner can interact with the implant magnet, resulting in complications for the HI recipient. This survey, which was conducted in two phases, aimed to evaluate the safety and performance of MRI scans for individuals with a HI manufactured by MED-EL (MED-EL GmbH, Innsbruck, Austria).

**Methods:**

A survey was developed and distributed in two phases to HEARRING clinics to obtain information about the use of MRI for recipients of MED-EL devices. Phase 1 focused on how often MRI is used in diagnostic imaging of the head region of the cochlear implant (CI) recipients. Phase 2 collected safety information about MRI scans performed on HI recipients.

**Results:**

106 of the 126 MRI scans reported in this survey were performed at a field strength of 1.5 T, on HI recipients who wore the SYNCHRONY CI or SYNCHRONY ABI. The head and spine were the most frequently imaged regions. 123 of the 126 scans were performed without any complications; two HI recipients experienced discomfort/pain. One recipient required reimplantation after an MRI was performed using a scanner that had not been approved for that implant. There was only one case that required surgical removal of the implant to reduce the imaging artefact.

**Conclusion:**

Individuals with either a SYNCHRONY CI or SYNCHRONY ABI from MED-EL can safely undergo a 1.5 T MRI when it is performed according to the manufacturer’s safety policies and procedures.

**Supplementary Information:**

The online version contains supplementary material available at 10.1007/s00405-020-06525-3.

## Introduction

Magnetic resonance imaging (MRI) is a commonly used, non-invasive imaging modality for diagnostics. Unlike other imaging modalities that are typically found in a radiology department, such as X-ray or computed tomography (CT), MRI scanners do not use ionizing radiation to obtain an image. Instead, MRI scanners use strong magnetic fields and radio frequency energy [1]. MRI is often the preferred imaging modality for the visualization and diagnosis of joint injuries that include damage to tendons and ligaments, damage to the spinal cord or nerves, intracranial tumors and lesions, and neurological diseases.

It was reported that 119 per 1000 people in Germany had an MRI scan between 2014 and 2018 [2]. The number of MRI scans being performed are continually increasing due to technological advancements and expanding indications. Unsurprisingly, hearing implant (HI) recipients are likely to require at least one MRI scan in their lifetime. Furthermore, with people receiving a HI at a younger age and considering the possibility of additional health complications that are associated with hearing loss, such as diseases of the inner ear, vestibular schwannomas, or neurofibromatosis 2 (NF2), the number of MRI scans that are required may be even higher [3]. Therefore, it is important to be aware of the safety of a HI recipient whilst undergoing an MRI scan.

An MRI scanner has a large magnetic coil that generates a strong, constant magnetic field. The strength of this magnetic field varies from one scanner to another, ranging from 0.2 T to 7.0 T or higher. Most radiology departments around the world have 1.5 T or 3.0 T MRI scanners, with stronger magnets resulting in higher resolution images [1, 4–7]. Safety is a key concern for HI recipients because a strong magnetic field can interact with the internal magnet of the HI and result in significant complications for the HI recipient [8]. It has been reported in the literature that HIs, such as cochlear implants (CIs), auditory brainstem implants (ABIs), middle ear implants, or bone conduction devices, are associated with unwanted complications during an MRI scan. These complications include large artefacts or distortions on the MR image, distortion or dislocation of the implant magnet, weakening of the implant magnet, demagnetization, or an increase in temperature in the vicinity of the implant [3, 9–17].

Shew et al. evaluated the safety of CI and ABI recipients during a 1.5 T MRI, and found that five out of 15 HI recipients experienced a complication [16]. Many HI recipients have also reported pain or discomfort during an MRI scan [11, 18]. In 2014, Kim et al. reported that out of 30 scans performed on 18 CI recipients, five recipients who wore head bandages during their MRI scan were unable to complete their scans due to pain; one recipient required surgical reimplantation due to magnet displacement of the implant [19]. One solution to this problem is to surgically remove the implant magnet before the MRI and surgically replace it afterwards. However, two additional operations carry the risk of infection, complications from anesthesia, damage to the HI, along with the additional costs involved with two surgical procedures [20, 21].

MED-EL (MED-EL GmbH, Innsbruck, Austria) have developed HIs that are suitable for use within an MRI scanner because removal of the implant magnet is not required. MED-EL received a CE mark in 2014 and FDA approval in 2015 for the SYNCHRONY to be used in an MRI up to 3.0 T, with subsequent regulatory approvals in other countries and regions. The SYNCHRONY has a diametrically magnetized and rotatable internal magnet that can turn freely and self-align in response to the external magnetic field. A diametrically magnetized internal magnet is less likely to result in the complications that were previously observed with many other HIs that use axially magnetized magnets [3, 16].

We distributed surveys in two phases to HEARRING clinics and collected data from the clinics on the use of MRI for HI recipients. The first phase aimed to evaluate how often MRI is used instead of other imaging modalities to image CI recipients and what local policies or procedures are in place for imaging CI recipients. The second phase aimed to retrospectively determine the number of complications reported from MED-EL HI recipients following an MRI scan. We hypothesize that no complications will be reported when MRI scans are performed according to the safety policies and procedures outlined by MED-EL [22], but that some complications may occur if these policies are not adhered to.

## Methods

### Phase 1

A survey was developed that consists of five questions about the diagnostic imaging modalities used for CI recipients. This survey was distributed to HEARRING clinics (see Appendix A) and each clinic was given two weeks to complete the survey. This survey was used to obtain information about the imaging modality that is most often used to image the head region of a CI recipient, how often radiologists refused to perform an MRI on a CI recipient, and if there were any local policies in place for imaging CI recipients.

### Phase 2

Following analysis of the results from the first survey, a second survey was distributed to the same HEARRING clinics to retrospectively collect information relating to MRI scans that were performed on recipients of MED-EL HIs (this included CI, ABI, middle ear implants, and bone conduction devices). The survey asked questions relating to the age of the HI recipient at the time of the MRI scan, the body region scanned, and the reason for the MRI. Information was also requested about the implant model, date of implantation, field strength of the MRI scanner, if a protective head bandage was used, and whether there were any complications during the MRI scanning procedure. The survey also asked if the imaging artefact affected the ability to diagnose the HI recipient and if users with a SYNCHRONY HI had the magnet removed prior to scanning.

Clinics were asked to complete the survey for every HI recipient that received an MRI scan between December 2013 and April 2018. This survey only collected anonymized data and information from health records available at each clinic. The survey could be completed online or completed offline and returned via email.

## Results

### Phase 1

Results from the first phase of the survey were collected from 26 HEARRING clinics, as shown in Table 1. The survey respondents at each participating clinic were CI surgeons currently active at the clinics and who reported on their experiences. The results showed that CT and MRI were both used to image the head region of CI recipients. Ten clinics stated that their decision to either use CT or MRI depended on the pathological investigation. Twelve clinics were aware of radiologists who refused to perform an MRI of the head region on a CI recipient and 12 clinics were unaware of such cases occurring. Of those clinics who were aware of radiologists who refused to perform MRIs on CI recipients, only three reported that radiologists were concerned about performing MRI scans on persons who were implanted with hearing devices. Nineteen clinics did not have a general policy for the removal of the CI’s internal magnet prior to imaging. Four clinics noted that their radiology department had the policy that CI recipients should typically be imaged using CT instead of MRI, whereas 19 clinics noted that this was not a policy in their radiology department.

**Table 1:** Results from Phase 1 of the survey. Twenty-six HEARRING clinics were asked how often MRI was used for cochlear implant (CI) recipients and whether local policies are in place at each clinic for performing an MRI on CI recipients

	Number of clinics
What do you typically do more of for imaging the head region?	
CT	8
MRI	3
CT and MRI	3
X-ray	1
It depends	10
No answer	1
Are you aware of radiologists who refuse to perform an MRI on people with a CI?	
Yes	12
No	12
Not anymore	1
No answer	1
If yes, was this because of a previous negative experience with people with a CI?	
Yes	3
No	6
No answer	3
In your local radiology department, is there a general policy on removing the implant magnet prior to an MRI?	
Yes	3
No	19
Yes/no	2
No answer	2
Is there a policy to image people with a CI with CT instead of MRI?	
Yes	4
No	19
Yes/no	2
No answer	1

### Phase 2

The second phase was completed by 17 of the 26 HEARRING clinics that received the survey. The results from this phase reported on the number of MRI scans that were performed on 88 recipients of MED-EL HIs between December 2013 and April 2018. This group of HI recipients consisted of 46 females and 41 males (one recipient’s gender was not reported). Seventy-eight recipients were unilaterally implanted and 10 were bilaterally implanted. Seventy-five recipients were adults (mean age at the time of MRI 55.6 years; standard deviation ± 18.0 years; range 18–83 years) and 10 were children (mean age at the time of MRI 5.4 years; standard deviation ± 5.8 years; range 1–17 years); the age of 3 recipients were not reported. The mean time period between implantation and receiving an MRI was 2.4 years (standard deviation ± 2.1 years; range 0.2–10 years).

Between December 2013 and April 2018, a total of 126 scans were performed at different field strengths on this group of 88 HI recipients. It should be noted that irrespective of whether recipients were uni- or bilaterally implanted, some recipients may have had more than one scan on their device(s): 73 HI recipients had one MRI scan (73 scans); eight had two scans (16 scans); four had three scans (12 scans); two had four scans (8 scans); and one HI recipient had five scans (5 scans). This accounts for 114 of the 126 reported scans. In terms of the scans performed on bilaterally implanted recipients, 8 recipients had only one scan (8 scans) and two recipients had two scans (4 scans), which accounts for the remaining 12 scans reported in the survey.

Table 2 provides a list of the type and model of HIs that the 88 recipients wore that resulted in the 126 scans reported in the survey. Table 2 also shows the number of times a type and model of the device was scanned at a particular magnetic field strength. The majority of HI recipients that underwent an MRI scan had a CI in one of the five models listed in the table; other HIs included the BONEBRIDGE BCI 601 bone conduction device, SOUNDBRIDGE VORP 503 middle ear implant, SYNCHRONY ABI, or other non-MED-EL implants (implanted on the contralateral side of bilateral HI recipients). A protective head bandage was used for 72 scans (i.e., 28 SYNCHRONY; 15 SYNCHRONY ABI; 20 CONCERTO; 9 SONATA; 4 BONEBRIDGE BCI 601; 3 PULSAR; 2 COMBI40 + ; 0 VORP 503; 2 other), not used for 23 scans, and it was not specified if a head bandage was used for 19 scans.

**Table 2:** Type and model of MED-EL hearing devices (MED-EL GmbH, Innsbruck, Austria) worn by the 88 HI recipients that were reported in Phase 2 of the survey

Type	Model	*N*_device_ (number of MRI scans per model of device)	*N*_T_ (number of MRI scans at different field strengths for each model of device)				
			0.2 T	1.5 T	3.0 T	Other	Unknown
Cochlear implant	COMBI40/COMBI40 +	4	0	3	1	0	0
	PULSAR	4	0	3	1	0	0
	SONATA	11	0	9	0	0	2
	CONCERTO	24	0	24	0	0	0
	SYNCHRONY	48	0	35	9	1	3
Bone conduction device	BONEBRIDGE BCI 601	6	0	4	0	2	0
Middle ear implant	SOUNDBRIDGE VORP 503	2	1	1	0	0	0
Auditory brainstem implant (ABI)	SYNCHRONY	24	0	24	0	0	0
Other implant*		3	0	3	0	0	0

*N*_device_ denotes the number of MRI scans that were performed on each model of the types of hearing devices reported in the survey. *N*_T_ denotes the number of MRI scans that were performed on each model of the types of hearing devices. Answer options consisted of magnetic field strengths 0.2, 1.5, and 3.0 T, and “other” and “unknown”; “other” was an answer option for cases where the field strengths were known to survey respondents but were not one of the numerical field strength options; “unknown” denotes field strengths that were not known to survey respondents

*Hearing implant from another company

Most of the MRI scanners reported in this survey had a 1.5 T coil (106 scans), 11 scanners had a 3.0 T coil, one had a 0.2 T coil, and eight were unknown or unspecified. This accounts for the 126 scans reported by survey respondents. Table 3 shows the regions that were imaged during each MRI scan and the reasons for performing the scan. The head and spine were the bodily regions that were scanned most frequently in HI recipients, with 77 scans of the head and 24 scans of the spine reported in the survey. The main reasons for performing the MRI scans were for monitoring NF2, neurological diseases, and for musculoskeletal/injury investigations.

**Table 3:** The region of the body that was imaged during each MRI scan and the reasons for performing the MRI scan

	*N*
Region of body imaged	
Head	77
Spine	24
Neck	5
Chest	2
Abdomen	6
Upper extremities	5
Lower extremities	9
Unspecified/unknown	1
Reasons for MRI	
NF2 monitoring	32
Neurological disease	20
Musculoskeletal/injury	15
Oncological	9
Pre-implantation of the second ear	6
Other	17
Unknown	15

For MRI scans of the head region, the artefact produced by the HI still allowed for diagnosis in 12 scans and limited diagnosis in 23 scans. Diagnosis was not possible for 13 scans and it was unknown if the artefact affected the diagnosis in 29 scans. For cases where the diagnosis was not possible, either a CT scan was performed, or an MRI scan was performed using a 1.5 T scanner instead of a 3.0 T scanner. The use of a scanner with a weaker magnetic field strength, i.e., 1.5 T instead of 3.0 T, reduced the artifact from the HI. Limited diagnosis was caused by distortion and susceptibility artefacts from the HI.

For users of the SYNCHRONY CI device, the internal magnet was only removed from one recipient prior to the MRI. In this case, the MED-EL magnet removal toolkit and non-magnetic spacer were not used. It was not reported whether the magnet was removed prior to the MRI for the other SYNCHRONY recipients.

In terms of other implants that this group of HI recipients had, it was reported that four scans were performed on three people that had another non-hearing implant. One had a hip implant (and underwent two MRI scans); one had titanium dental implants (one MRI scan); and one had an unknown additional implant (one MRI scan).

### Adverse events

During two different MRI scanning events, HI recipients reported discomfort and unexpected sensations. Both users were bilaterally implanted. Recipient #1 wore a SONATA on the right side and a non-MED-EL implant on the left. Recipient #2 wore a PULSAR on the right side and a COMBI40 + on the left. It was noted that the pain was related to the non-MED-EL side (left side) for Recipient #1, but it was not specified if the sensations were restricted to one side or on both sides for Recipient #2. One of the scans was aborted after the discomfort was reported. All devices worked properly afterwards without any further complications.

During a 0.2 T MRI scanning event, dislocation of the floating mass transducer (FMT) was observed for one SOUNDBRIDGE VORP 503 middle ear implant recipient after the MRI was performed outside of the recommended safety policies and procedures; this implant has not been approved for a 0.2 T MRI. The user was a unilaterally implanted male adult undergoing an MRI of the head region. A head bandage was not used during the scan. The recipient also reported a lack of auditory sensations after the MRI. The recipient was later reimplanted and the implant was returned to the manufacturer for further evaluation, where it was found that there was no damage to or weakening of the FMT magnet.

No adverse events were reported during the other 123 MRI scans reported during this investigation.

## Discussion

The results of Phase 1 of the survey were obtained from 26 HEARRING clinics and showed that CT and MRI were both used to image the head region of CI recipients. Twelve clinics reported that they were aware of radiologists who refused to perform an MRI on a CI recipient; however, only three clinics had a negative experience while performing an MRI on a CI recipient. It is thought that many radiology departments are unfamiliar with the differences between the various types of HI devices and are not aware of which implants are compatible with MRI. Therefore, further information should be distributed to radiologists regarding the MRI safety and compatibility of implants from different manufacturers. In 19 of the 26 clinics that participated in this survey, there were no policies in place regarding the removal of the CI’s internal magnet prior to MRI scanning or that CI recipients should receive a CT scan instead of an MRI scan. The results of this survey led to the development of a second survey that aimed to collect further information relating to all MED-EL HIs and to evaluate their safety and performance during an MRI scan.

Phase 2 of the survey showed that 126 MRI scans were performed on 88 HI recipients in 17 HEARRING clinics between December 2013 and April 2018. The majority of the HI recipients (73 out of 88) required only one MRI over this timeframe, however, there were some HI recipients who required up to five scans. It is interesting to note the distribution of HI devices in terms of the number of MRI scans that were performed. Out of 123 scans that were performed on different types and models of MED-EL HIs, 48 scans were performed on the SYNCHRONY CI and 24 scans were performed on the SYNCHRONY ABI. The remaining 3 scans were performed on HIs from other manufacturers. The majority of scans (106 out of 126) were performed at 1.5 T. Eleven scans were performed at 3.0 T, of which 9 out of the 11 scans were performed on SYNCHRONY CI recipients. The results of our two-phase survey are thus most indicative of the frequency of use and safety of MRI scans at a field strength of 1.5 T when the HI recipient wears either the SYNCHRONY CI or the SYNCHRONY ABI. Only one scan performed at a field strength of 0.2 T was reported. This seems to indicate that 0.2 T MR imaging of HI recipients is no longer routinely performed.

A cadaveric study previously demonstrated that 25% of implants (4 out of 16) moved during a 1.5 T MRI when performed without a compressive head wrap, compared to 0 of 16 when a compressive head wrap was used [23]. In our survey, most of the clinics reported that they used a protective head bandage during MRI scans, even though it was not a safety requirement. Assuming that the MRI is performed according to the manufacturers’ safety policies and procedures, the use of a head bandage is optional for the following MED-EL implants: SYNCHRONY, SYNCHRONY 2 (including PIN and ABI variants), SYNCHRONY ST, BONEBRIDGE BCI 601, BONEBRIDGE BCI 602, and SOUNDBRIDGE VORP 503. All other models from MED-EL require a simple elastic head bandage that should be wrapped around the head at least 3 times. The bandage should sit firmly on the head but should not cause any pain to the HI recipient.

Sixty percent of MRI scans reported in our survey were performed to image the head region, with the spine being the second most common region scanned. The distribution of MRI scans taken over various regions of the body are similar to those published by Grupe et al. [8] and Walker et al. [15]. The most common reasons to perform an MRI on a HI recipient were for monitoring NF2, neurological diseases, and for muscloskeletal/injury investigation. It is important to note that this survey only pertained to the health records of HI recipients that were available at each clinic. There may have been additional MRI scans of other body regions, such as the knee, or for unrelated conditions that were performed in outpatient centers that were not included here.

For MRI scans of the head region, the imaging artefact still allowed for a full diagnosis, or for a limited diagnosis in the majority of cases. When the diagnosis was not possible, CT imaging or MRI using a lower field strength (i.e., 1.5 T instead of 3.0 T) were performed. Similar results have been published in other studies, including studies with HIs from other manufacturers [12, 15, 16]. The mean artefact size was previously reported as 7.43 cm along the long axis and 4.16 cm along the short axis [19], with more pronounced artefacts observed with a 3.0 T MRI scanner in comparison to a 1.5 T MRI [24]. For cases where MRI is required to evaluate brain pathologies on the implanted side of a HI user, it may be possible to reduce the artefact by selecting a suitable MRI sequence with an optimized set of parameters and artefact reduction sequences. If this is not sufficient and the imaging artefact still affects the diagnosis, then it is possible to surgically remove the implant magnet [24, 25].

The SYNCHRONY HI received a CE mark (in 2014) and FDA approval (in 2015) for use in an MRI scanner with field strengths up to 3.0 T. There have only been a few reported cases where the removal of the internal magnet was necessary to reduce the imaging artefact [26, 27]. To the best of our knowledge, no complications following the removal of the SYNCHRONY magnet have been reported. According to our survey respondents, the magnet was only removed from one SYNCHRONY HI recipient prior to an MRI and no adverse events were reported. This is in agreement with published reports that the freely rotating magnet in the SYNCHRONY allowed recipients to undergo an MRI without experiencing pain or discomfort, and without magnet dislocation or demagnetization, even at 3.0 T [10, 16, 28]. Nonetheless, if it is required to remove the magnet from a SYNCHRONY implant prior to an MRI (e.g., due to the presence of an imaging artefact in the region of interest), then it should be possible to do so safely [26, 27].

Individuals who require regular MRI surveillance of particular regions of the head should be carefully counselled prior to receiving a HI. It is recommended that individuals who require regular MRI surveillance of regions of the head should receive a HI with a removable magnet, such as the SYNCHRONY, which allows for the easier removal and reinsertion of the implant magnet. Helbig et al. [27] reported a study involving a young child with bilateral SYNCHRONY implants. The child required magnet removal prior to an MRI so that a case of hydrocephalus could be investigated. The total time required for two surgical procedures was 1 h 45 mins or 3 h 15mins including the MRI scan; there were no complications reported during the entire procedure.

Our survey found that a large majority of MRI scans were performed on MED-EL HI recipients without any complications. There were no dislocations or weakening of the implant magnet when the MRI was performed while following MED-EL’s safety policies and procedures [22]. Only one case was reported in our survey where a surgical procedure was required for reimplantation of a device: a recipient of the SOUNDBRIDGE VORP 503 implant was scanned with a 0.2 T MRI even though this middle ear implant has only been approved for use at 1.5 T and not at 0.2 T. Todt et al. reported that a 1.5 T MRI was performed on SOUNDBRIDGE VORP 503 users. All scans were performed without any degree of pain, change in pure tone audiometry results, or audio processor fittings [34]. Several studies have documented cases in which recipients of the SOUNDBRIDGE had an MRI at a magnetic field strength for which the SOUNDBRIDGE is not approved, yet no adverse events or complications were reported [16, 29–31]. Nonetheless, there have been many reports of adverse events, where HI recipients of devices by other manufacturers experienced pain, magnet dislocations, heating of the implant, induction of electrical current that damaged or resulted in malfunctioning of the implant, or demagnetization of the internal HI magnet [3, 12, 15–17, 19, 32, 33]. Having said that, there were no reports of damage to hearing nerves, loss in the ability to hear with the HI after the MRI, or long-term pain or uncomfortable sensations from any MED-EL HI recipients after the MRI in any of the clinics who completed our survey. Previous studies reported pain as the most common complication during an MRI for HI users [3, 8], with clinicians often having to stop the MRI scan due to the level of pain experienced [19]. There were only two cases reported in our survey where the HI recipients experienced discomfort or unexpected sensations during the MRI; there were no further complications after the MRI for either of these individuals.

Many studies have been published about the outcomes of MRI in individuals with a CI from Cochlear Ltd. (Sydney, Australia), however, outcomes with CIs from other manufacturers and other HIs such as ABIs, middle ear implants, or bone conduction devices are not often reported. In our survey, we found no reports of complications with ABI devices. Only one complication was observed with a middle ear implant whereby the MRI scan was performed with a field strength of 0.2 T, for which the implant has not received approval. Therefore, it is important for clinics and radiology departments to perform MRIs in accordance with the safety policies and procedures that have been released by each manufacturer to avoid unnecessary adverse events or additional surgical procedures for the HI recipient.

### Limitations and future studies

As previously discussed, this investigation involved the distribution of surveys and the retrospective collection of data from the health records of HI recipients that were available within each HEARRING clinic, and as such, there may have been additional MRI scans performed in outpatient centers that were not included here. Furthermore, due to the nature of an investigation that relies on survey responses that are based on health records, it is possible for some inaccuracies to have been reported. The responses to our survey are indicative of the frequency of use and safety considerations pertaining to 1.5 T MRI scans that are routinely performed on recipients of SYNCHRONY CIs and SYNCHRONY ABIs. Only a small number of responses pertained to field strengths other than 1.5 T (in particular, 0.2 T and 3.0 T), other MED-EL devices, and devices from other manufacturers. Thus, we cannot draw any reliable conclusions on those scenarios based on the survey responses that were collected. Nonetheless, the data obtained is still valuable and useful for developing strategies to create greater awareness of the safety issues relating to MRI scanning of HI recipients: the data is indicative of where radiology departments might lack the knowledge and experience in performing MRIs at different field strengths on different types of HIs from different manufacturers. Three survey responses selected the field strength option “Other”, which indicated that it was known to the survey respondent that the field strength used was other than the answer options 0.2, 1.5, and 3.0 T. It would have been of interest to know what those field strengths were and what the radiologists’ reasons were for scanning at those field strengths. This raises the question of the routine use of other field strengths, particularly those between 1.5 and 3.0 T (e.g., 2.5 T), and radiologists’ knowledge, experience, and confidence in MR imaging of HI recipients at those field strengths. Survey design should therefore provide respondents with the possibility to elaborate on their answers as much as possible.

Future studies might consider investigating the ability to visualize the internal acoustic canal, cerebellopontine angle, or other features of the inner ear from an MRI scan of a HI recipient—this would be particularly interesting when following individuals with vestibular schwannomas or NF2. Small studies have been performed on this topic [35], but a large-scale study covering a wide range of HIs would be invaluable.

## Conclusion

The safety of the HI recipient during an MRI scan is a priority for clinics across the world. Results of this survey, which was conducted in two phases, found that individuals with either the SYNCHRONY CI or SYNCHRONY ABI, both devices manufactured by MED-EL, can safely undergo an MRI at a field strength of 1.5 T provided that the scan is performed according to the manufacturer’s safety policies and procedures. Therefore, radiology departments should become familiar with these and be willing to perform an MRI on a SYNCHRONY CI or ABI recipient when required.

## Supplementary Information

Below is the link to the electronic supplementary material.

Supplementary file1 (DOCX 19 KB)
