# Supplementary material for: Two-phase survey on the frequency of use and safety of MRI for hearing implant recipients
Source: Eur Arch Otorhinolaryngol. 2021 Mar 31;278(11):4225–33. doi: 10.1007/s00405-020-06525-3 (PMC8486706; doi:10.1007/s00405-020-06525-3)
Supplement: Supplementary file 1 — Supplementary file1 (DOCX 19 KB) [file 405_2020_6525_MOESM1_ESM.docx]

**Appendix A: List of HEARRING clinics that participated in the two-phase survey**

Córdoba Sanatorium Allende, Servicio de Otorrinolaryingologia (Servicio ORL), Córdoba, Argentina

University of Western Australia, Crawley, Perth, Australia

Ear Sciences Center, Lions Hearing Clinic, Subiaco, Australia

ENT Department of Medical University of Innsbruck, Innsbruck, Austria

St. Pölten University Hospital, St. Pölten, Austria

Vienna Medical University – General Hospital AKH, Vienna, Austria

ENT Department, Antwerp University Hospital (UZA), Edegem/Antwerp, Belgium

Hospital for Rehabilitation of Cranio-Facial Anomalies, Bauru-Sao Paulo, Brazil

London Health Sciences Center – University Hospital, London, Ontario, Canada

Centre Hospitalier Universitaire (CHU) de Rennes, Rennes, France

Bochum St. Elisabeth University Hospital, Bochum, Germany

Universität Rostock “Otto Körner”, Klinik und Poliklinik für Hals-Nasen-Ohrenheilkunde, Rostock, Germany

Würzburg ENT University Hospital, Würzburg, Germany

Shinshu University School of Medicine, Matsumoto, Japan

Institute of Sensory Organs, Kajetany, Nadarzyn, Poland

Department of Teleaudiology and Screening, World Hearing Center of the Institute of Physiology and Pathology of Hearing, Kajetany, Nadarzyn, Poland

St. Petersburg ENT and Speech Research Institute, St. Petersburg, Russia

King Abdullah Ear Specialist Center, King Saud University, Riyadh, Saudi Arabia

Madrid Hospital La Paz, Madrid, Spain

Karolinska University Hospital, Solna, Sweden

Department for ENT, Head and Neck Surgery, Bern University Hospital, Bern, Switzerland

Bradford Royal Infirmary Yorkshire Auditory Implant Center, Bradford, United Kingdom

Pediatric ENT Department, Royal Manchester Children’s Hospital, Manchester University NHS Foundation Trust, Manchester Academic Health Science Centre, Manchester, United Kingdom

Division of Infection, Immunity and Respiratory Medicine, Faculty of Biology, Medicine and Health, University of Manchester, Manchester, United Kingdom

Kansas University Center for Hearing and Balance Disorders, Department of Otorinolaryngology, Kansas City, USA

UNC Ear and Hearing Center at Chapel Hill School of Medicine, Chapel Hill, North Carolina, USA
